# Sr-doped surfaces with 2D black phosphorus nanosheets for enhanced photothermal antibacterial activity and zirconia implant osseointegration

**DOI:** 10.1093/rb/rbaf033

**Published:** 2025-04-26

**Authors:** Huan Cheng, Jiaquan Chen, Yan Wang, Yinyan Zhang, Tianyun Qin, Haobo Sun, Wen Si, Ningyao Sun, Yingyue Sun, Lifeng Xiong, Zhennan Deng, Lei Lu, Peng Gao, Jinsong Liu

**Affiliations:** School and Hospital of Stomatology, Wenzhou Medical University, Wenzhou 325027, China; School and Hospital of Stomatology, Wenzhou Medical University, Wenzhou 325027, China; School and Hospital of Stomatology, Wenzhou Medical University, Wenzhou 325027, China; School and Hospital of Stomatology, Wenzhou Medical University, Wenzhou 325027, China; School and Hospital of Stomatology, Wenzhou Medical University, Wenzhou 325027, China; School and Hospital of Stomatology, Wenzhou Medical University, Wenzhou 325027, China; School and Hospital of Stomatology, Wenzhou Medical University, Wenzhou 325027, China; School and Hospital of Stomatology, Wenzhou Medical University, Wenzhou 325027, China; School and Hospital of Stomatology, Wenzhou Medical University, Wenzhou 325027, China; School and Hospital of Stomatology, Wenzhou Medical University, Wenzhou 325027, China; School and Hospital of Stomatology, Wenzhou Medical University, Wenzhou 325027, China; School and Hospital of Stomatology, Wenzhou Medical University, Wenzhou 325027, China; School and Hospital of Stomatology, Wenzhou Medical University, Wenzhou 325027, China; School and Hospital of Stomatology, Wenzhou Medical University, Wenzhou 325027, China

**Keywords:** dental implants, zirconia, black phosphorus, Sr, antibacterial photothermal therapy, osteogenesis

## Abstract

Zirconia (ZrO_2_) has emerged as a preferred material for dental implants due to its excellent chemical inertness, absence of metal allergies and esthetic appeal. However, its limited bioactivity regarding infection resistance and early osseointegration hinders its implantation success rate compared to titanium implants. Herein, we developed a PDPA@Sr/BP coating for ZrO_2_ implants to address these limitations. First, inspired by the adhesive properties of mussel foot proteins, a PDPA@Sr coating enriched with positively charged amine groups and strontium (Sr) ions was applied to the ZrO_2_ surface. This coating stably anchored black phosphorus (BP) to the implant, effectively regulating its degradation rate and ensuring long-lasting antibacterial properties. Under near-infrared (NIR) light irradiation, BP generated localized heat, efficiently killing bacteria. Simultaneously, the release of Sr and phosphate ions from the PDPA@Sr/BP coating promoted bone formation and enhanced osseointegration. This study systematically evaluated the antibacterial effects and osseointegration-promoting properties of the PDPA@Sr/BP coating through both *in vitro* and *in vivo* experiments. The results demonstrated that compared to untreated ZrO_2_ surfaces, the coating significantly enhances the implant’s antibacterial properties and accelerates its surface osseointegration. This study proposes an innovative strategy to improve the clinical performance of ZrO_2_ implants, demonstrating substantial potential for clinical translation.

## Graphical abstract

**Figure:**
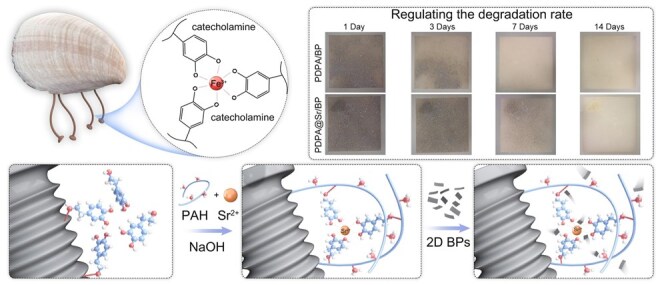

## Introduction

Dental implants are widely regarded as the primary solution for tooth restoration due to their excellent functionality and esthetics. Zirconia (ZrO_2_) has attracted considerable attention among emerging materials due to its exceptional mechanical strength [1, 2], chemical inertness [3], corrosion resistance and color resemblance to natural teeth [4], making it highly desirable in clinical applications. Despite these advantages, ZrO_2_ exhibits limited bioactivity in the early stages post-implantation, particularly regarding infection resistance and bone integration [5]. Insufficient osteogenic activity can significantly prolong the healing time required for stable integration of ZrO_2_ implants with newly formed bone. During this phase, small gaps between the implant and bone tissue create an ideal bacterial invasion and inflammation niche. The oral microbiome comprises numerous pathogenic bacteria that can accumulate on implant surfaces, promoting biofilm formation [6]. Additionally, studies have shown that *Porphyromonas gingivalis* can contribute to the degradation of ZrO_2_, thereby reducing its mechanical properties [7]. Common clinical interventions, such as mechanical debridement, ultrasonic cleaning, laser therapy and local or systemic antibiotic administration, are widely employed to eliminate pathogenic bacteria on implant surfaces. However, they frequently result in significant patient trauma and often demonstrate limited efficacy. Therefore, it is essential to develop noninvasive antibacterial strategies with low reliance on antibiotics that also promote osteogenesis for bioinert ZrO_2_ implants [8].

While electricity, magnetism and ultrasound-sensitive antibacterial materials possess noninvasive and antibiotic-free characteristics, near-infrared (NIR) responsive materials offer additional advantages. In addition to their capacity to synergistically eliminate bacteria through heat and reactive oxygen species [8, 9], NIR-responsive materials enhance local tissue blood circulation and promote osseointegration of implants [10, 11], making them particularly suitable for applications in dental implant restoration. Currently, gold, silver and copper nanoparticles are classic photothermal antibacterial materials, leveraging localized surface plasmon resonance to convert light into heat, thereby killing bacteria [12, 13]. However, in some cases, these metal nanoparticles exhibit relatively low photothermal conversion efficiency, requiring extended periods to generate sufficient heat for effective antibacterial action [14]. Moreover, metal nanoparticles may accumulate in the body, raising concerns about potential toxicity, particularly with long-term use, which poses significant biosafety challenges [15]. As an alternative, 2D photothermal antibacterial coatings have garnered significant attention in recent years [16, 17]. These coatings exhibit high NIR photothermal conversion efficiency, promote cell adhesion and bone mineralization and hold great promise in bone tissue engineering applications [18, 19]. However, many 2D materials (e.g. graphene) are inherently dark in color, which may compromise the esthetics of dental restorations, particularly when combined with ZrO_2_ [20]. Additionally, these coatings are prone to burst release of their components or loaded drugs *in vivo*, and their rapid dispersion and clearance may lead to localized high concentrations and potential cytotoxicity [21], thereby limiting their therapeutic efficacy and clinical applicability.

Black phosphorus (BP) is a novel 2D material with exceptional photothermal properties, characterized by a layered crystal structure similar to graphite. Through exfoliation techniques, BP can be produced in nanosheet form with varying thicknesses [22]. Unlike the zero-bandgap semiconductor graphene, BP exhibits a layer-dependent bandgap ranging from 0.3 eV (bulk) to approximately 2.0 eV (monolayer) [23]. It demonstrates broad absorption across the visible spectrum [24], which grants it notable NIR photothermal characteristics for photothermal therapy (PTT) [25, 26]. With its excellent photothermal conversion efficiency and large specific surface area [27], BP is a promising candidate for photothermal agents. Additionally, compared to other 2D materials like graphene and MXenes, BP degrades into phosphate ions (${\text{PO}}_{4}^{3-}$) [28, 29]—essential elements for the human skeletal system—providing versatile raw materials for bone regeneration [30]. BP’s biodegradable byproducts can transform into calcium phosphate nanoparticles, promoting bone regeneration through *in situ* biomineralization, and showcasing excellent biocompatibility and osteogenic potential [18, 31, 32]. Notably, extensive research has focused on BP in titanium implants [33, 34], while its application in zirconia implants remains limited. More importantly, the biodegradable nature of BP ensures that its dark color does not have a long-term impact on esthetic appearance. However, the chemically inert surface of ZrO_2_ lacks reactive functional groups, posing a challenge in anchoring BP to the ZrO_2_ implant surface and regulating its degradation rate.

**Figure 1.**
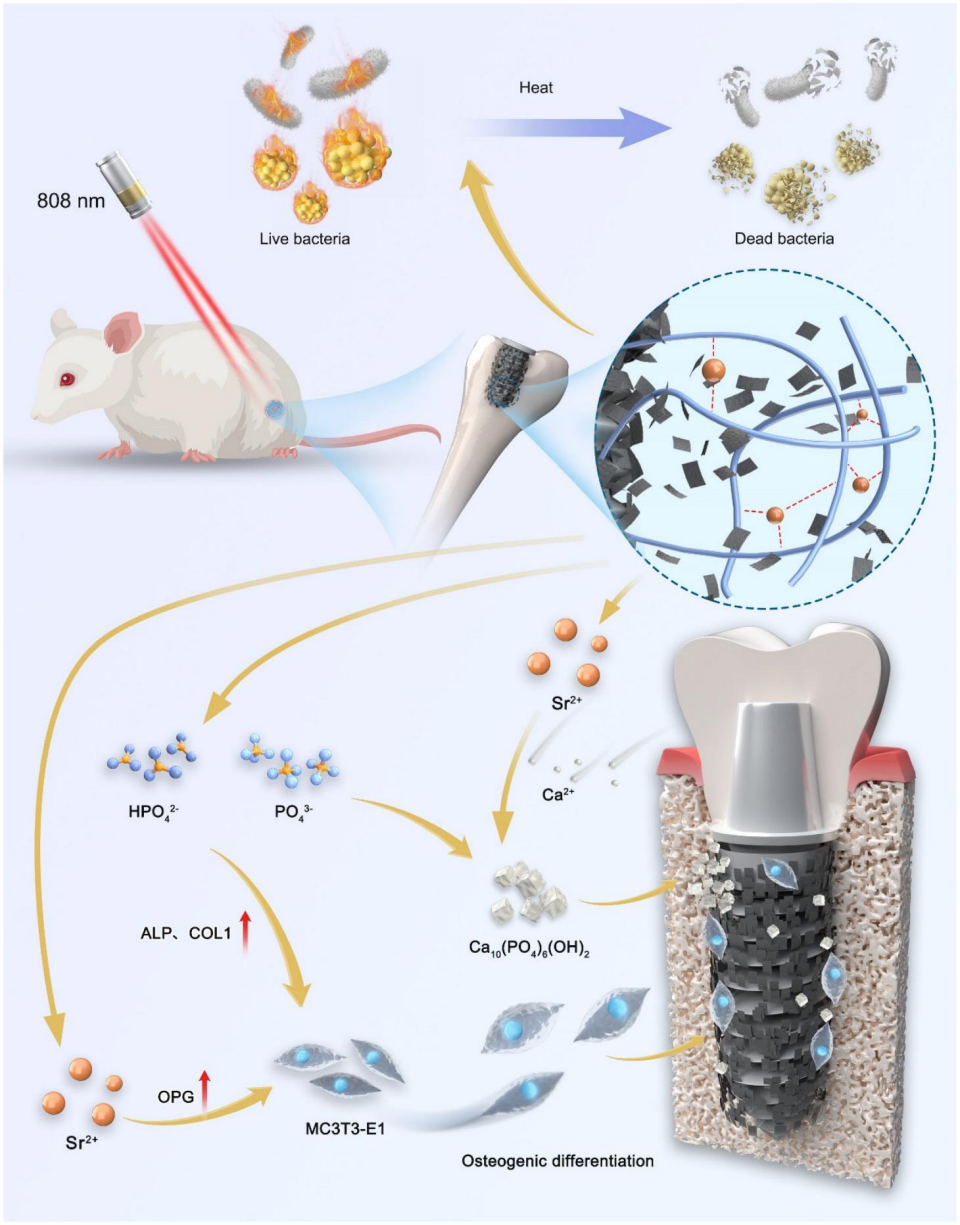
Schematic illustration of photothermal antibacterial activity and osteoinductive effects of PDPA@Sr/BP coating.

Based on the negative charge and coordination capability of BP nanosheets (BPNs), we designed a PDPA@Sr coating enriched with Sr ions and primary amine groups, which can effectively load BPNs for efficient antibacterial activity and enhance osseointegration of implants (Figure 1). Primarily, owing to the abundant amine groups and Sr ions on its surface, the PDPA@Sr coating effectively stabilizes BPNs on the implant surface via electrostatic adsorption and coordination chemistry, concurrently serving a crucial role in regulating BP degradation. Second, under 808 nm laser irradiation, the PDPA@Sr and BPNs synergistically generate high temperatures, exhibiting excellent antibacterial properties. More importantly, the sustained release of Sr ions from the coating, along with ${\text{PO}}_{4}^{3-}$ generated from BP degradation, promotes rapid osseointegration of the implant. This study systematically evaluated the formation mechanism of the novel PDPA@Sr/BP coating, as well as its antibacterial efficacy and osseointegration-promoting properties through *in vitro* and *in vivo* experiments.

## Materials and methods

### Materials and reagents

BP crystal (99.998%) was purchased from Zhongke Experimental Materials Co., Ltd (China) and preserved in an inert atmosphere. Dopamine, polyallylamine hydrochloride (PAH), anhydrous strontium chloride (SrCl_2_) and sodium hydroxide (NaOH) were purchased from Macklin Co., Ltd (China). Tris-HCl (≥ 99%) was purchased from Sigma-Aldrich Co., Ltd (USA). Acid orange II was purchased from CATO Co., Ltd (China). The α-MEM medium was purchased from Gibco (Thermo Fisher Scientific Co., Ltd, USA). Fetal bovine serum (FBS) was purchased from Vivacell Co., Ltd (Germany). Antibiotic mixture was purchased from New Cell & Molecular Biotech Co., Ltd (China). FITC-phalloidin and DAPI were purchased from Solarbio Co., Ltd (China). Cell Counting Kit-8 (CCK-8) was purchased from Beyotime Co., Ltd (China).

### Samples preparation

#### Preparation of BPNs

BPNs were synthesized via liquid-phase exfoliation using BP dispersed in N-methylpyrrolidone (NMP) at a concentration of 0.2 mg/ml. The dispersion was subjected to sonication with an ultrasonic cell pulverizer (Xiaomei XM150T, Jiangsu, China) for 1 h, using a duty cycle of 3 s on and 2 s off, in an ice bath. This was followed by additional sonication using an ultrasonic cleaner for 20 min, repeated over a total of nine cycles. The resulting suspension was centrifuged at 4000 rpm to remove precipitates, and the supernatant was further centrifuged at 13 500 rpm, with the precipitate being retained. The precipitate was then washed with anhydrous ethanol and resuspended. The final dispersion was stored at 4°C in a sealed container.

#### Preparation and pretreatment of ZrO_2_ substrate

Commercially available 3 mol% Y_2_O_3_-stabilized tetragonal zirconia polycrystals were first sectioned into disks with dimensions of 10 mm × 10 mm × 1.25 mm using a diamond wire saw (STX-202A, Kejing, China). The specimens were then sintered in a high-temperature furnace (inFire HTC SPEED, Sirona, Germany). The sintering protocol consisted of heating at a rate of 4°C/min to 1480°C with a 2-h holding time, followed by cooling at 6°C/min to 800°C. After sintering, the final dimensions of the zirconia disks were 8 mm × 8 mm × 1 mm, and these were designated as the ZrO_2_ group. A subset of the zirconia samples (for EDS analysis) was further polished using a polishing machine (UNIPOL-802, Kejing, China) equipped with 400-grit silicon carbide abrasive disks at a speed of 100 rpm under continuous water cooling for 15 min, in order to achieve a smoother surface.

These samples were placed face-up in a petri dish and fully immersed in a 1 mg/ml dopamine solution (1 mM Tris, pH = 8.5) under light-protected conditions for 24 h. Following this initial immersion, the solution was discarded, and the samples were subjected to a second immersion under identical conditions for the same duration. Upon completion of the second immersion, the samples underwent ultrasonic cleaning with anhydrous ethanol, acetone and deionized water, followed by a drying process.

#### Preparation of PDPA@Sr/BP

The samples prepared as described were immersed in a solution containing PAH (0.4% v/v) and NaOH (3 mg/ml). Following a 12 h soaking period, these treated samples were designated as the PDPA group. For another set of samples, an equal volume of SrCl_2_ (20 mg/ml) solution was added to the same PAH and NaOH solution mixture, and the samples were similarly soaked for 12 h. These samples were referred to as the PDPA@Sr group. After soaking, all samples were subjected to ultrasonic cleaning, dried and stored for future use. Subsequently, the previously prepared BPNs suspension (0.5 mg/ml) was separately introduced to the PDPA and PDPA@Sr samples. Using a vacuum pressure drying chamber, the BPNs were loaded onto the samples through strong electrostatic interactions over a duration of 6 h. These samples were designated as the PDPA/BP group and PDPA@Sr/BP group, respectively. Upon completion of the preparation process, the samples were stored under vacuum, shielded from light and maintained in a dry environment for future use.

### Sample characterization

The surface morphology and elemental composition of samples were assessed through the utilization of transmission electron microscope (TEM, FEI, USA), scanning electron microscopy (SEM, Nova Nano SEM450, USA) and energy-dispersive X-ray spectroscope (EDS, Nova Nano SEM450, USA). Surface compositions and the valence states of elements were determined using X-ray photoelectron spectroscopy (XPS, Escalab 250Xi, USA), while the zeta potential and size of nanoparticles were gauged using a Malvern particle size analyser (Zetasizer Nano, UK). The water contact angle (WAC) was measured employing a contact angle goniometer (SDC-200S, Sindin, China). Tensor II (Bruker, Germany) was used to detect the Fourier Transform Infrared Spectroscopy (FTIR) of BPNs. To evaluate the photothermal performance of PDPA@Sr/BP, an 808-nm NIR laser with a power density of 1.0 W/cm^2^ was used, and the temperature changes were recorded using an infrared thermal imaging camera (FLIR E6390, USA).

### Quantitative analysis of amino groups

Each sample group was immersed in 500 µl of Acid Orange II (AO II) aqueous solution (2 mg/ml, pH 5) for 4 h, followed by ultrasonic cleaning with dilute hydrochloric acid solution (pH 5) three times to remove loosely adsorbed AO II from the sample surface. Subsequently, the samples were immersed in 500 µl of sodium hydroxide solution (0.4 mg/ml, pH 12) to elute the AO II adsorbed on the sample surface into the solution. Quantitative analysis was performed at OD 485 nm using a microplate reader.

### Degradation and release of Sr and P

Samples were immersed in 5 ml of ultrapure water. At designated intervals of 1, 3, 7 and 14 days, the samples were transferred to fresh deionized water. Subsequently, the collected liquids were analysed using inductively coupled plasma mass spectrometry (ICP-MS, Agilent 7500, Agilent).

### Experiment on the interaction between Sr^2+^ and BPNs

Tris solution (1 ml, pH 8.5) was added to 5 ml of BPNs suspension (1 g/ml). Under continuous stirring, 1 ml of anhydrous SrCl_2_ solution was then added, and stirring was maintained for 12 h. After this stirring period, the mixture was allowed to stand for 30 min, during which photographs of the sample were taken. The mixed solution was subsequently centrifuged at 8000 rpm for 20 min to remove the supernatant. This centrifugation process was repeated three times, after which the zeta potential of the final sample was measured.

### *In vitro* bacteriological experiment

*Staphylococcus aureus* (*S. aureus*) and *Escherichia coli* (*E. coli*) were cultured in Luria-Bertani (LB) medium at 37°C for 24 h to reach a density of 1 × 10^5^ colony-forming units (CFU) per ml. Subsequently, 500 μl of the bacterial suspension was inoculated onto five different samples (circular, diameter = 10 mm): ZrO_2_, PDPA, PDPA@Sr, PDPA/BP and PDPA@Sr/BP + NIR (1.0 W/cm^2^). Following a 10 min treatment, an agar plate assay was performed to evaluate bacterial survival and density after a 16 h incubation period. After identical treatment, the bacteria on the sample surfaces were fixed using 2.5% glutaraldehyde, followed by ethanol gradient dehydration and solvent exchange. The samples were then introduced into the FE-SEM chamber for electron imaging. Following identical treatment, bacteria on the sample surfaces were eluted via ultrasonic treatment, and the resulting suspensions were centrifuged at 3000 rpm for 5 min to collect the bacterial cells. The collected bacteria were then stained with the fluorescent nucleic acid dye SYTO-9 to label live bacteria, while propidium iodide (PI) was used to stain dead bacteria. The bacterial samples were incubated with the staining solution at room temperature in the dark for 30 min. Finally, 1–2 μl of the bacterial suspension was placed on a microscope slide and examined using an inverted fluorescence microscope. For each sample, images were captured from five different fields of view, and ImageJ software was used to calculate the live/dead bacteria ratio based on the fluorescence images.

### *In vitro* evaluation of the osteogenic performance of the material

#### Cell culture

Mouse embryonic osteoblast precursor cells (MC3T3-E1) were cultured in a complete medium consisting of 89% α-MEM, 10% FBS and 1% antibiotic mixture (100 IU/ml penicillin and 100 μg/ml streptomycin). The cells were incubated in a humidified atmosphere of 5% CO_2_ at 37°C, with the medium refreshed every 3 days. For subsequent biological assays, MC3T3-E1 cells were seeded onto membranes with a diameter of 12 mm at a density of 1 × 10^4^ cells per membrane, using 24-well tissue culture plates as holders. To ensure statistical reliability, four parallel replicates of each sample were prepared for each experiment.

#### Live/dead cell staining assay

In this experiment, MC3T3-E1 cells were seeded at a density of 2 × 10^4^ cells/well and co-cultured with the samples in α-MEM medium. Fluorescence microscopy was performed on day 1. After removing the original medium, a staining solution containing calcein-AM (for live cells) and PI (for dead cells) (Live/Dead Cell Staining Assay Kit, YEASEN, China) was added, and the cells were incubated at room temperature for 5 min. Fluorescence images were captured from five random fields per group, and the live/dead cell ratio was quantified using ImageJ software based on the fluorescence signals.

#### Cell morphology

MC3T3-E1 cells were seeded onto various sample surfaces at a density of 2 × 10^4^ cells per well and cultured at 37°C in a 5% CO_2_ atmosphere for 3 days. Following incubation, the cells were washed three times with PBS and subsequently fixed. To increase membrane permeability, 0.1% Triton X-100 was applied. The cytoskeleton was stained with FITC-phalloidin for 2 h, and the nuclei were stained with DAPI for 5 min. Fluorescence images were randomly captured to evaluate cell morphology.

#### Cell viability assay

MC3T3-E1 cells were seeded onto various sample surfaces at a density of 2 × 10^4^ cells/well and cultured at 37°C in a 5% CO_2_ atmosphere for 1 and 3 days. After incubation, the viability of MC3T3-E1 cells on the surfaces of the different sample groups was assessed using the CCK-8 assay (NCM Biotech, China), following the manufacturer’s instructions.

#### Cell BCIP/NBT ALP staining assay

MC3T3-E1 cells were seeded onto various sample surfaces at a density of 2 × 10^4^ cells/well and cultured at 37°C in a 5% CO_2_ atmosphere for 4 and 7 days. Following incubation, the cells were washed three times with PBS and subsequently fixed. To increase membrane permeability, 0.1% Triton X-100 was applied. Alkaline phosphatase (ALP) activity was assessed using the AKP assay kit (Jiancheng, Nanjing, China) and the BCA protein assay kit (Beyotime, China), following the manufacturer's instructions.

#### Extracellular matrix mineralization assay

MC3T3-E1 cells were seeded onto various sample surfaces at a density of 2 × 10^4^ cells/well and cultured at 37°C in a 5% CO_2_ atmosphere for 21 days, and then the cells were washed with PBS for three times and fixed. To stain the mineralized nodules, 500 μl of alizarin red staining (ARS, Solarbio, China) was added and incubated for 30 min. Images were captured at four random locations to evaluate mineralization. Subsequently, the mineralized nodules were immersed in 500 μl of a 10 wt% cetylpyridinium chloride solution and shaken at room temperature for 15 min. The absorbance of dissolved calcified nodules was measured at an optical density of 540 nm.

#### Assessment of gene expression related to cell mineralization

MC3T3-E1 cells were seeded onto various sample surfaces at a density of 2 × 10^4^ cells/well and cultured at 37°C in a 5% CO_2_ atmosphere for 4 and 7 days, and then the cells were washed with PBS for three times. Total RNA was extracted using an extraction kit (Tiangen, China) following cell lysis in trizol. The concentration of RNA was measured using a NanoDrop 2000 Spectrophotometer (Thermo Fisher Scientific, USA). Complementary deoxyribonucleic acid (cDNA) was synthesized using the PrimeScriptTM RT Reagent kit (Perfect Real Time, Takara, Japan). To analyse the expression levels of genes such as ALP, collagen type I (COL1), osteocalcin (OCN), osteopontin (OPN), glyceraldehyde-3-phosphate dehydrogenase (GAPDH) was used as an internal reference. The primer sequences for the target genes are listed in Supplementary Table S1.

### *In vivo* experiments

#### Preparation of implants

The implant screws were fabricated using the same yttria-stabilized zirconia (YSZ) material as in the previous experiments, by proportionally downsizing clinical dental implant designs and performing precision machining with a milling device (imes-icore 350i, imes-icore, Germany). The resulting samples were then sintered following the same procedure as described in the *in vitro* experiments and subjected to identical surface modification. The final screws, measuring 10 mm × 3 mm with a thread pitch of approximately 1 mm and a thread depth of about 0.4 mm, were used for subsequent *in vivo* studies.

#### In vivo antibacterial activity evaluation

The experimental protocol was approved by the Institutional Animal Care and Use Committee of Wenzhou Institute, University of Chinese Academy of Sciences (approval number: WIUCAS24111101). For *in vivo* antibacterial study, 16 Sprague Dawley (SD) rats housed under uniform conditions were used. The rats were randomly divided into two major groups: the *S. aureus* group and *E. coli* group. Each group was further divided into two subgroups: NIR (−) and NIR (+), with four rats per group.

Following the described method, the rats were anesthetized with isoflurane, and the hair on their backs was shaved. The skin was disinfected with povidone-iodine. Incisions were made using a scalpel, and samples from each group were placed subcutaneously. Then, 50 μl of *S. aureus* (1 × 10^8^ CFU/ml) or 50 μl of *E. coli* (1 × 10^8^ CFU/ml) was injected onto the surface of the samples. For the NIR (+) group, NIR irradiation (1 W/cm^2^, 808 nm) was applied 1 day after sample implantation for 60 s, maintaining a temperature of approximately 42°C. The NIR (−) group did not receive additional treatment. Three days later, the rats were euthanized by an intraperitoneal injection of an overdose of pentobarbital sodium. The samples were retrieved and placed in sterile 24-well plates. Each well received 300 μl of sterile PBS, and the samples were subjected to ultrasonic treatment for 5 min. The sonicated solution containing dislodged bacteria was collected, and bacterial counts were performed using the plate counting method to evaluate the antibacterial efficacy of each sample group against both bacteria.

The skin covering the sample area was excised, fixed in 4% paraformaldehyde and processed for paraffin embedding. Hematoxylin and eosin (H&E) staining was used to assess inflammatory infiltration, and immunohistochemistry was performed to detect TNF-α to evaluate the inflammatory response in the epidermis.

#### In vivo osteogenic performance evaluation

The implant screws, with a diameter of 1.2 mm and a length of 10 mm, were obtained after sintering according to previously described procedures, followed by surface modification as described earlier. In this experiment, 16 eight-week-old female SD rats, weighing between 195 and 235 g, were used. The rats were obtained from Zhejiang Vital River Experimental Animal Technology Co., Ltd, China. The rats were randomly divided into four groups, with four rats per group. Inhalation anesthesia was performed using isoflurane (oxygen flow rate: 1.5 l/min, concentration: 1%). The hair around both knees of the rats was shaved, and the skin was disinfected using povidone-iodine. A distal femoral incision was made using a No. 11 scalpel blade, and sequential drilling was performed using 0.8-, 1.0- and 1.2-mm drills under continuous irrigation with 4°C saline. The previously prepared implants were then inserted, and the surgical site was sutured using 3-0 sutures. Postoperatively, penicillin was administered for three consecutive days to prevent infection, and non-cyclic irradiation of the implant sites was performed every three days.

One month after implantation, the rats were euthanized. The femurs were harvested and fixed in 4% paraformaldehyde. After decalcification, the femurs were processed for paraffin embedding. H&E staining was performed to evaluate new bone formation around the implants and Masson’s trichrome staining was used to assess the formation of collagen fibers surrounding the implants.

### Statistical analysis

All experiments in this study were conducted with a minimum of three repetitions. The experimental data were recorded as the mean ± standard deviation. If the data followed a normal distribution, statistical analysis was given by the one-way analysis of variation, considering values of *P* < 0.05 a significant difference.

## Results

### Characterization of BPNs and PDPA@Sr coating

**Figure 2.**
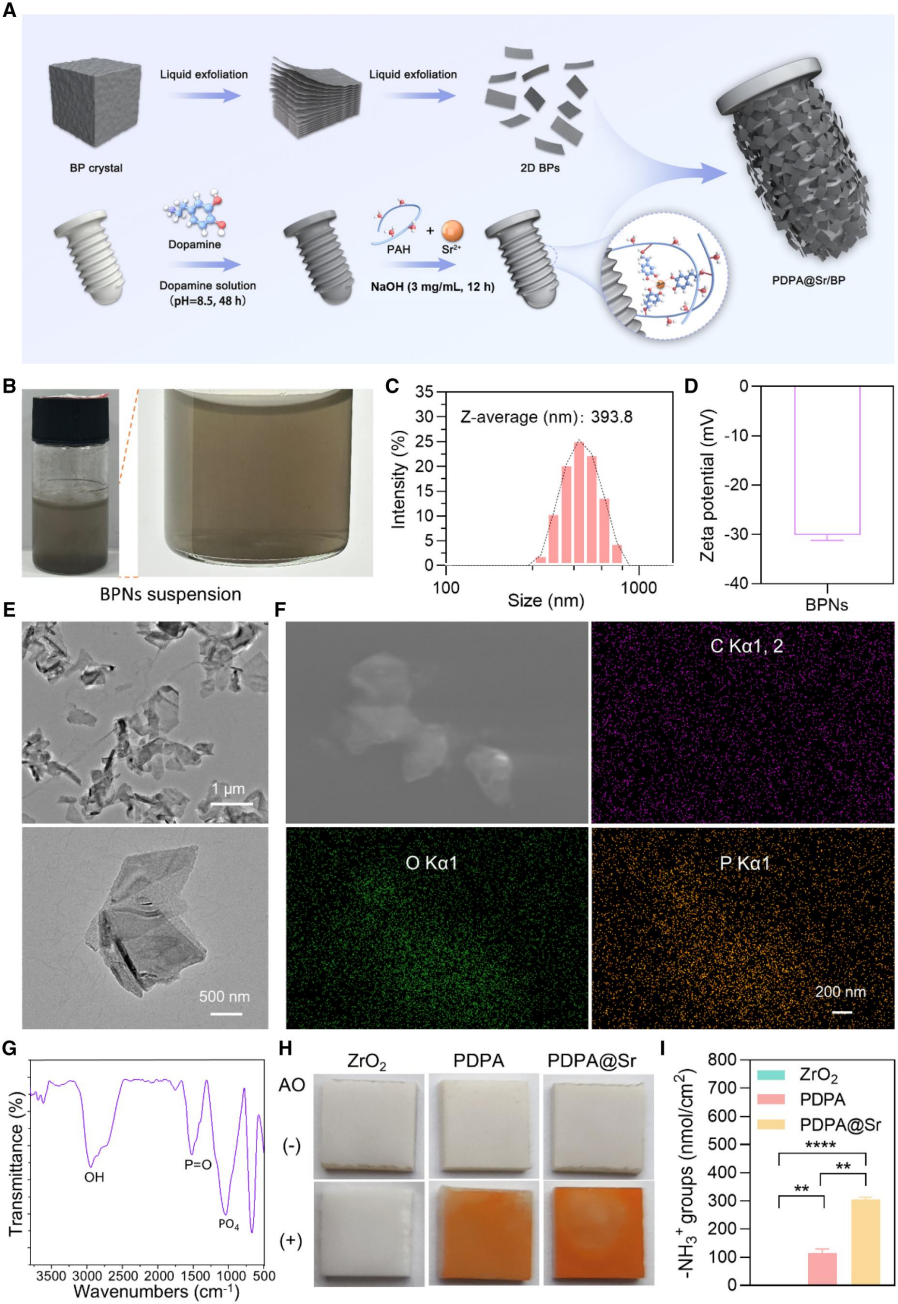
Preparation and characterization of BPNs. (**A**) Schematic illustration of the synthesis procedures for PDPA@Sr/BP. (**B**) Images of BPNs suspension after exfoliation. (**C**) Particle size distribution of BPNs. (**D**) Zeta potential of BPNs suspension. (**E**) TEM images of BPNs. (**F**) SEM and EDS images of BPNs. (**G**) FTIR spectrum of BPNs powder. (**H**, **I**) Acid orange staining images of the sample surface and quantitative analysis of −${\text{NH}}_{3}^{+}$ groups (**P < 0.01 and ***P < 0.001, *n* = 3).

The exfoliated 2D BPNs were immobilized onto the ZrO_2_ surface using a mussel-inspired coating enriched with amino groups and Sr ions (Figure 2A). To control the oxidation of BP crystals during exfoliation, we employed a liquid-phase exfoliation method using NMP as the solvent [31], successfully preparing a well-dispersed solution of BPNs (Figure 2B). Following exfoliation, we replaced the organic solvent NMP with double-distilled water and analysed the BPNs using a Malvern particle size analyser. Dynamic light scattering (DLS) measurements indicated that the particle size distribution of the BPNs was predominantly between 100 and 1000 nm, with an average particle size of 393.8 nm, confirming successful nanoscale exfoliation (Figure 2C). Subsequently, we measured the zeta potential of the BPNs. As shown in Figure 2D, the zeta potential of the exfoliated BPNs was approximately −30 mV, consistent with previously reported values for BPNs [35]. TEM results revealed that multiple ultrasonication cycles yielded BPNs with a controllable size and thickness (Figure 2E). EDS was used to characterize the elemental composition of the BPNs, showing the presence of phosphorus (P) and oxygen (O) elements on the BPNs (Figure 2F). Additionally, the FTIR results indicated that BPNs were successfully obtained, as evidenced by the appearance of P=O and P–O_4_ peaks, further confirming that partial oxidation of the BPNs has occurred (Figure 2G). Oxidation of BPNs typically leads to structural degradation, initiating a decomposition process. However, this degradation also ensures the long-term biocompatibility of BPNs without compromising the esthetic properties of ZrO_2_, making these nanosheets highly suitable for surface modification of ZrO_2_ implants.

To firmly immobilize BPNs onto the ZrO_2_ implant surface, we first prepared a polydopamine (PD) coating with strong adhesive properties on the smooth ZrO_2_ surface. Subsequently, the PD coating was reconstructed *in situ* by disrupting its non-covalent bonds with a strong alkaline solution, while simultaneously incorporating Sr^2+^ and PAH which contains abundant ethylamine groups. This resulted in the formation of a PDPA@Sr coating enriched with Sr^2+^ and –${\text{NH}}_{3}^{+}$ groups on the surface. Then we used negatively charged acid orange II dye to semi-quantitatively assess the surface positive charge of the PDPA@Sr coating (Figure 2H). The results showed that the ZrO_2_ group surface exhibited almost no adsorption of acid orange II, while the PDPA group adsorbed approximately 100 nmol/cm^2^ of the acid orange II, and the PDPA@Sr group reached around 300 nmol/cm^2^ (Figure 2I). Therefore, it can be inferred that the surface of the PDPA@Sr coating is abundant in –${\text{NH}}_{3}^{+}$ groups.

### Characterization of PDPA@Sr/BP coating

**Figure 3.**
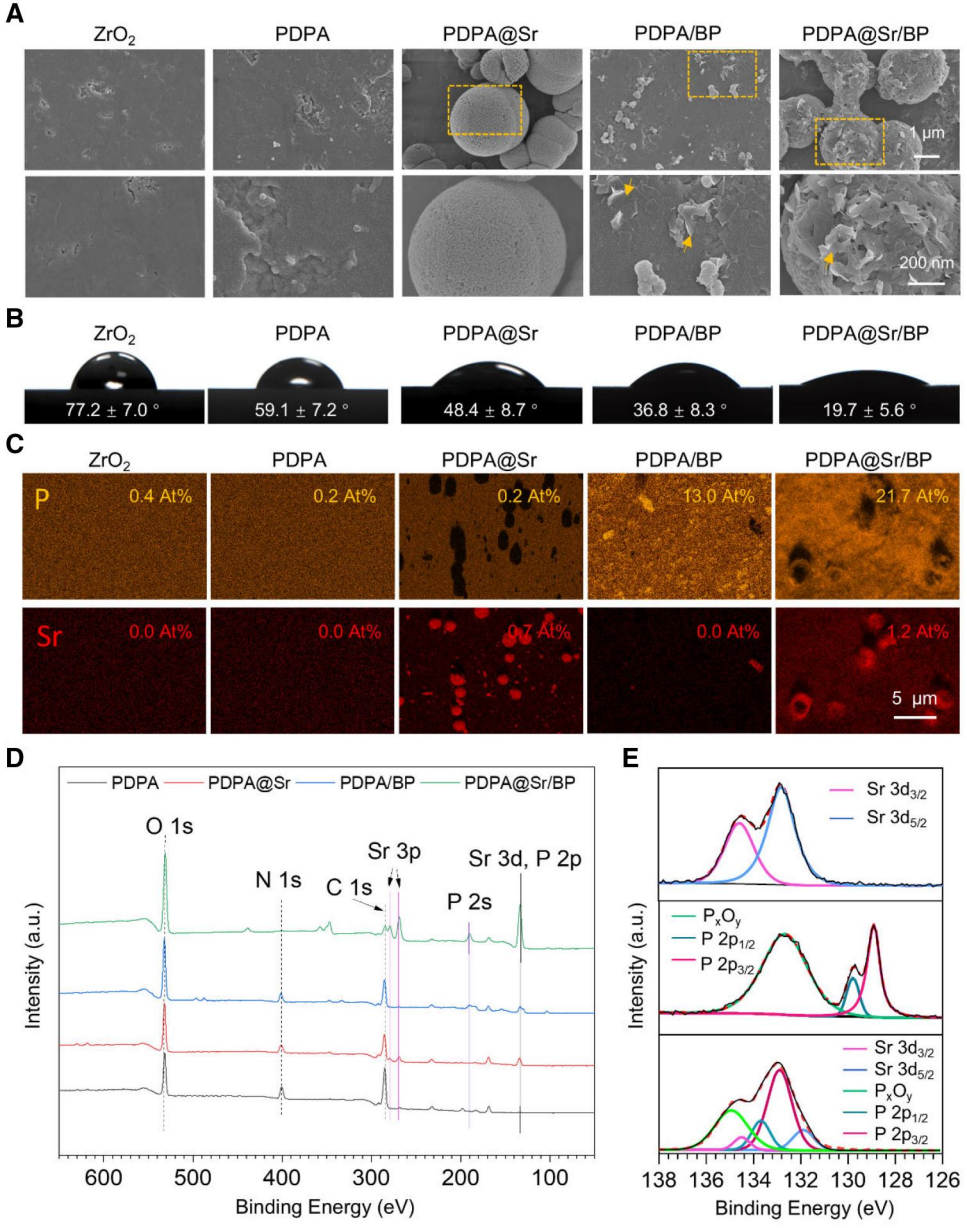
Characterization of PDPA@Sr/BP coating. (**A**) SEM images, (**B**) water contact angle and (**C**) EDS images of sample surfaces. (**D**) XPS spectra of sample surfaces. (**E**) High-resolution XPS spectra of Sr 3d and P 2p in PDPA@Sr, PDPA/BP and PDPA@Sr/BP.

Through the electrostatic immobilization of BPNs with highly negative zeta potential onto the PDPA@Sr surface, the PDPA@Sr/BP coating was successfully formed. We then characterized the surface morphology of each sample group (Figure 3A). The ZrO_2_ group exhibited only minor surface indentations, while samples modified with PDPA showed the presence of some aggregated nanoparticles. In comparison, a large number of smooth micro- and nano-scale microspheres were observed on the PDPA@Sr surface. On both the PDPA/BP and PDPA@Sr/BP surfaces, a significant number of BPNs were clearly visible. Additionally, the PDPA@Sr/BP surface was noticeably rougher than the smooth microspheres on the PDPA@Sr surface, likely due to the coverage by BPNs. The presence of Sr-containing particles and the adsorption of BP both increase the surface roughness. As surface hydrophilicity plays a crucial role in regulating cellular behavior and influencing osseointegration [36], we further evaluated the WAC of the sample surfaces (Figure 3B). The WAC of the unmodified ZrO_2_ group was 77.2 ± 7.0°, indicating weak hydrophilicity. While surfaces modified with Sr^2+^ and BPNs exhibited significantly improved hydrophilicity, with the PDPA@Sr/BP coating showing the smallest WAC at 19.7 ± 5.6°, demonstrating the highest hydrophilicity. EDS results (Figure 3C) were analysed to evaluate the elemental composition of the samples. The ZrO_2_ group and PDPA-modified samples showed minimal P and Sr elements on the surface. In contrast, the Sr-doped groups exhibited significant Sr element distribution in spherical forms. These forms resembled the microspheres observed in the SEM images, confirming that they were likely Sr^2+^ microspheres adsorbed on the sample surface. Moreover, the EDS map of the BP-added groups revealed a broad distribution of P elements, suggesting that the two-dimensional nanosheets observed in the SEM images were indeed BPNs. Notably, as shown in Supplementary Figure S1, the O element distribution on the PDPA/BP sample surface was slightly higher than in the previous three groups. The higher O distribution indicates some degree of oxidative degradation of BP in the PDPA/BP group. However, in comparison to the PDPA/BP group, the O distribution on the PDPA@Sr/BP sample surface was significantly reduced. The Sr and P elemental distributions in PDPA@Sr/BP reached 1.2 and 21.7 at%, respectively, noticeably higher than in the Sr^2+^- and BP-doped groups. Although we cannot conclude that more Sr^2+^ and BP were adsorbed on the PDPA@Sr/BP sample surface, we can at least infer that the oxidation degree of BP on the PDPA@Sr/BP surface may be lower compared to the PDPA/BP coating. This suggests that the stability of BP may have been improved after Sr^2+^ doping.

Subsequently, XPS analysis was performed on each sample group modified with PDPA (Figure 3D). All four sample groups displayed a C 1s peak near 285 eV, an N 1s peak around 400 eV and an O 1s peak at approximately 530 eV. Additionally, PDPA@Sr displayed Sr 3p double peaks around 270 eV and Sr 3d peaks near 130 eV, while PDPA/BP showed P 2s and P 2p peaks around 190 eV, indicating the successful binding of individual Sr^2+^ or BPNs to the PDPA coating. PDPA@Sr/BP displayed both Sr 3p double peaks and Sr 3d peaks, as well as P 2s and P 2p peaks. Notably, due to the proximity of the peak positions, the Sr 3d doublet and P 2p doublet in PDPA@Sr/BP appeared as a single peak. We conducted peak fitting on the high-resolution XPS spectra of Sr 3d and P 2p around 130 eV for PDPA@Sr, PDPA/BP and PDPA@Sr/BP (Figure 3E). In the PDPA@Sr spectrum, two double peaks were fitted at approximately 132 and 134 eV, with an intensity ratio close to 3:2, consistent with the spin-orbit splitting characteristics of Sr 3d_5/2_ and Sr 3d_3/2_ peaks. In the PDPA/BP spectrum, the P 2p_3/2_ and P 2p_1/2_ peaks were fitted around 128 and 129 eV, respectively, with an intensity ratio of 2:1. Additionally, a single peak around 133 eV was attributed to the P_*x*_O_*y*_ peak, indicating partial oxidation of BP based on previous studies. After peak fitting in the high-resolution spectrum of PDPA@Sr/BP, the binding energies of the peaks shifted: the Sr 3d doublet shifted to lower binding energy, while the P 2p doublet shifted to higher binding energy, resulting in overlapping Sr 3d and P 2p peaks due to their proximity. Given the relatively high electronegativity of P in BP, it is plausible that partial negative charges or lone electron pairs form on the surface, potentially interacting electrostatically with Sr^2+^ and forming coordination bonds. This context could explain the peak shifts in Sr 3d and P 2p observed in XPS spectra, along with the previously observed potential change after mixing BPNs with Sr^2+^. We deduced that BPNs binding to the PDPA@Sr coating surface may involve coordination with Sr^2+^, contributing to these spectral shifts.

### BP degradation behavior and photothermal effect of PDPA@Sr/BP coating

**Figure 4.**
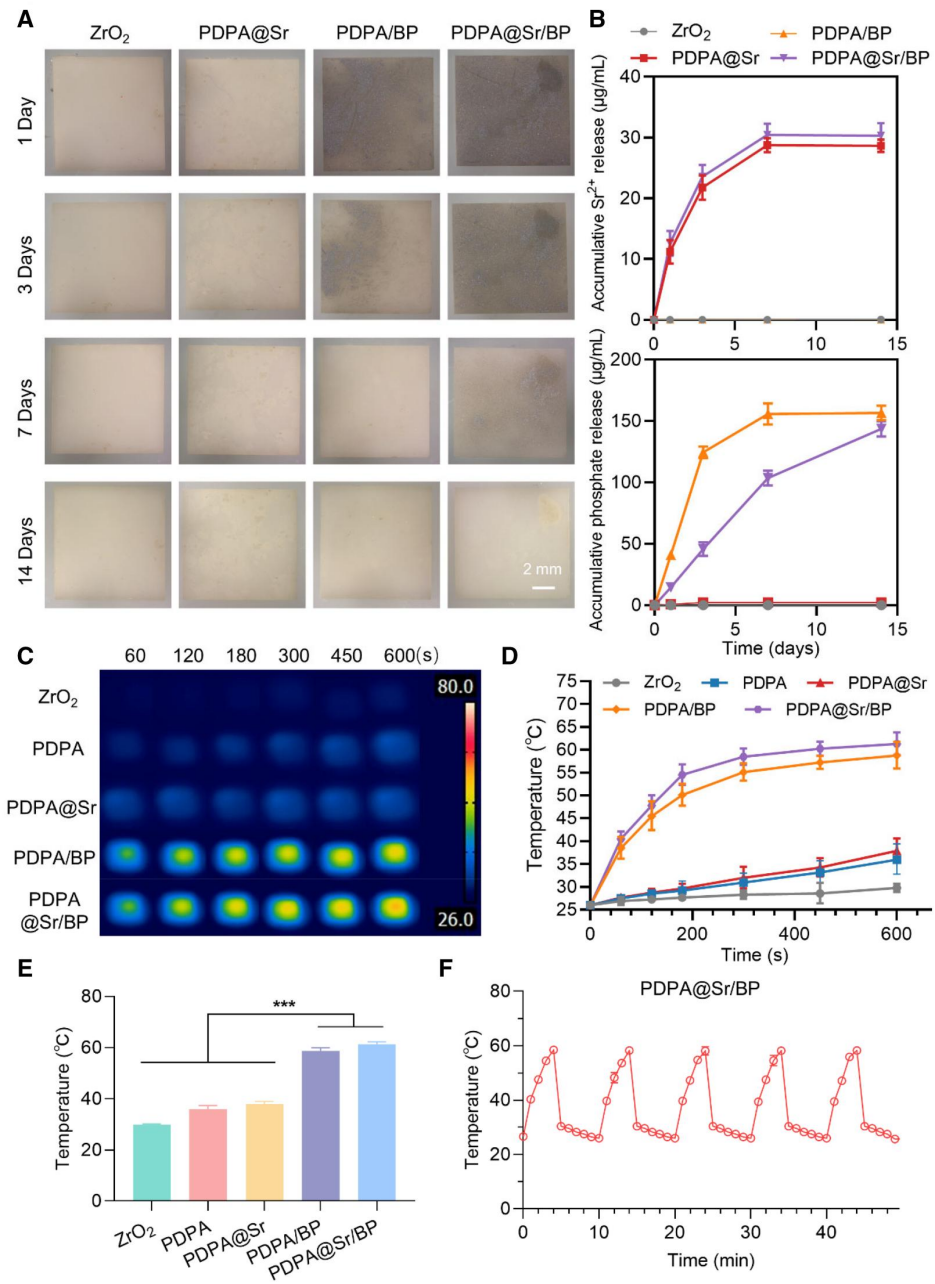
BP degradation behavior and photothermal effect of PDPA@Sr/BP coating. (**A**) Surface images of samples after immersion in ultrapure water for 1, 3, 7 and 14 days. (**B**) Accumulative phosphate release measured (μg/ml). (**C**, **D**) Thermal images and corresponding photothermal curves under NIR exposure in PBS. (**E**) Maximum surface temperature of each sample after 600 s. (**F**) Temperature profile of PDPA@Sr/BP over five NIR on/off cycles (***P < 0.001, *n* = 3).

Since BP degrades into ${\text{PO}}_{4}^{3-}$ and hydrogen phosphate ions (${\text{HPO}}_{4}^{2-}$) in the physiological environment, these ions are essential components in bone mineralization [37]. ${\text{PO}}_{4}^{3-}$ combines with calcium (Ca^2+^), providing the raw material for the formation of hydroxyapatite (Ca_10_(PO_4_)_6_(OH)_2_), which is the main component of bone matrix mineralization [38]. The gradual degradation of BP provides a stable and continuous source of P, promoting bone formation and repair [39]. The release of Sr^2+^ also contributes to osteoblast proliferation and differentiation to some extent, while inhibiting osteoclast activity [40, 41]. Additionally, during bone mineralization, strontium ions can partially replace Ca^2+^ and participate in the formation of hydroxyapatite [42–44]. This substitution does not affect the crystal structure of hydroxyapatite; rather, it enhances the mechanical strength and biological stability of bone, promoting the formation of mineralized bone. Therefore, the release of Sr^2+^ and ${\text{PO}}_{4}^{3-}$ may have a synergistic effect, effectively promoting osteogenesis on the surface of the implant *in vivo*. We conducted elemental release tests for Sr and P in each sample group and photographed the surface of the samples at each time point (Figure 4A). As shown, the surface color of the ZrO_2_ and PDPA@Sr groups remained almost unchanged over time, while the PDPA/BP and PDPA@Sr/BP groups, which adsorbed BP, initially displayed a distinct and uniform black color. Notably, the black color of the PDPA/BP surface faded significantly over time, becoming nearly indistinguishable from the other two groups at one-week time point. In contrast, the black color of the PDPA@Sr/BP group faded more slowly, remaining uniform after three days and retaining some black coverage even at the one-week time point. Although some black material residue remained on the PDPA@Sr/BP surface at the two-week time point. According to the ICP-MS results for Sr and P element release (Figure 4B), only the PDPA@Sr and PDPA@Sr/BP samples containing Sr^2+^ showed detectable Sr^2+^ release. On day 1, the release reached 11.283 and 12.346 μg/ml, respectively, and by day 3, the levels were 21.918 and 23.245 μg/ml. Both samples peaked on day 7 and remained stable until day 14, with levels of 28.460 and 30.356 μg/ml, respectively. For P element release, only the BP-containing samples (PDPA/BP and PDPA@Sr/BP) showed detectable levels. The values for PDPA/BP on days 1, 3 and 7 were 43.138, 122.552 and 153.965 μg/ml, stabilizing at day 14. However, the release levels for PDPA@Sr/BP were lower in the first seven days, with values of 14.043, 45.474 and 104.011 μg/ml, respectively, but the gap narrowed over time. By day 14, the P release for PDPA@Sr/BP reached 140.699 μg/ml, indicating that BPNs continued to degrade over this period, consistent with the earlier surface image results. Analysing the P degradation curves for PDPA@Sr/BP, it can be seen that the degradation rate remained fairly constant for the first seven days, with only a slight slowdown observed at day 14. This suggests that the degradation of BPNs on the PDPA@Sr/BP surface proceeded at a relatively steady pace. The slower degradation of BPNs on the PDPA@Sr/BP surface compared to PDPA/BP could be due to an interaction between Sr^2+^ and BPNs. To explore this possibility, we added SrCl_2_ to a stirred solution of BPNs in Tris buffer (pH 8.5) and observed the results. As shown in Supplementary Figure S2, in comparison to the previously shown BPNS suspension, the addition of Sr^2+^ induced significant aggregation of BPNs. Zeta potential measurements showed a potential of −0.8 mV, suggesting that coordination or adsorption between Sr^2+^ and BPNs may have occurred, possibly shielding the negative charges on the BPNs surface. This suggests that when BPNs are adsorbed onto the PDPA@Sr surface, a similar coordination or electrostatic shielding effect may occur. Some studies have shown that coordination or electrostatic adsorption involving BPNs can enhance their stability by masking active sites, thereby slowing their degradation [45]. Additionally, the PDPA@Sr surface, which is rich in positively charged –${\text{NH}}_{3}^{+}$ groups, may also contribute to the slower degradation of BPNs on the PDPA@Sr/BP surface.

Furthermore, the degradation rate of BPNs plays a critical role in both antibacterial performance and bone integration. A rapid degradation rate facilitates the immediate release of ${\text{PO}}_{4}^{3-}$, which can accelerate hydroxyapatite formation and early-stage bone mineralization. However, excessive degradation may lead to a sudden surge in ion concentration, potentially disrupting local ionic homeostasis and impairing cellular adaptation over time. In contrast, a slower degradation profile ensures a sustained supply of phosphate ions, supporting long-term osteoblast activity and continuous mineralization while minimizing the adverse effects associated with abrupt ion release. Additionally, the photothermal properties of BPNs contribute to their antibacterial function. Rapid degradation may lead to the premature loss of BPNs, limiting their photothermal efficiency. In contrast, a more gradual degradation allows BPNs to retain their photothermal capacity for an extended period, ensuring a prolonged antibacterial effect under NIR irradiation. This sustained photothermal antibacterial activity can effectively reduce infection risks at the implant interface, providing a favorable environment for bone regeneration. However, we speculate that PDPA@Sr/BP has limited scratch resistance, which may negatively impact implantation. Overall, BPN degradation kinetics influence osteogenic ion release, antibacterial efficacy and osseointegration. Optimizing the degradation rate can balance bioactivity, antimicrobial protection and bone repair. Future research will focus on refining this behavior to enhance its synergy in bone regeneration and antimicrobial applications while improving the scratch resistance of PDPA@Sr/BP.

Due to the high photothermal conversion efficiency of BPNs and the inherent photothermal properties of PDPA, the newly designed PDPA@Sr/BP composite coating demonstrates exceptional photothermal conversion efficiency. As shown in Figure 4C and E, under 808 nm NIR irradiation (1.0 W/cm^2^, 10 cm), the temperature of PDPA@Sr/BP soaked in PBS rapidly increased from 25°C to approximately 62°C within 100 s, and after 10 min of continuous irradiation, the temperature stabilized at 61.3°C. In comparison, other groups modified with PDPA coatings exhibited moderate photothermal conversion, reaching approximately 40°C after 100 s of irradiation. However, unmodified ZrO_2_ showed significantly lower photothermal conversion efficiency, with only a 7.8°C increase after 10 min of irradiation under the same conditions. This indicates that both PDPA and BP play critical roles in enhancing the photothermal conversion efficiency of the coating. Furthermore, to assess the laser-induced photothermal stability of PDPA@Sr/BP, cyclic temperature changes were measured under the same NIR laser parameters (Figure 4F). PDPA@Sr/BP exhibited excellent photothermal stability, with no significant degradation observed during five laser on/off cycles. Over the 50-min period, with five heating and cooling cycles, the maximum temperature consistently reached around 60°C, highlighting the durability of PDPA@Sr/BP for continuous photothermal bacterial ablation applications.

In subsequent experiments, as a temperature range of 40–42°C is generally known to promote tissue repair and healing [39], an irradiation temperature of 42°C was selected to balance effective biofilm ablation with the protection of surrounding normal tissues and cells. Based on preliminary experiments, the temperature was precisely controlled at approximately 42°C during each non-cyclic NIR irradiation (1 W/cm^2^, 808 nm) for 60 s. This temperature was found to effectively eradicate bacteria while ensuring the safety of surrounding tissues and cells, making it a promising strategy for PTT.

### *In vitro* photothermal antibacterial properties

**Figure 5.**
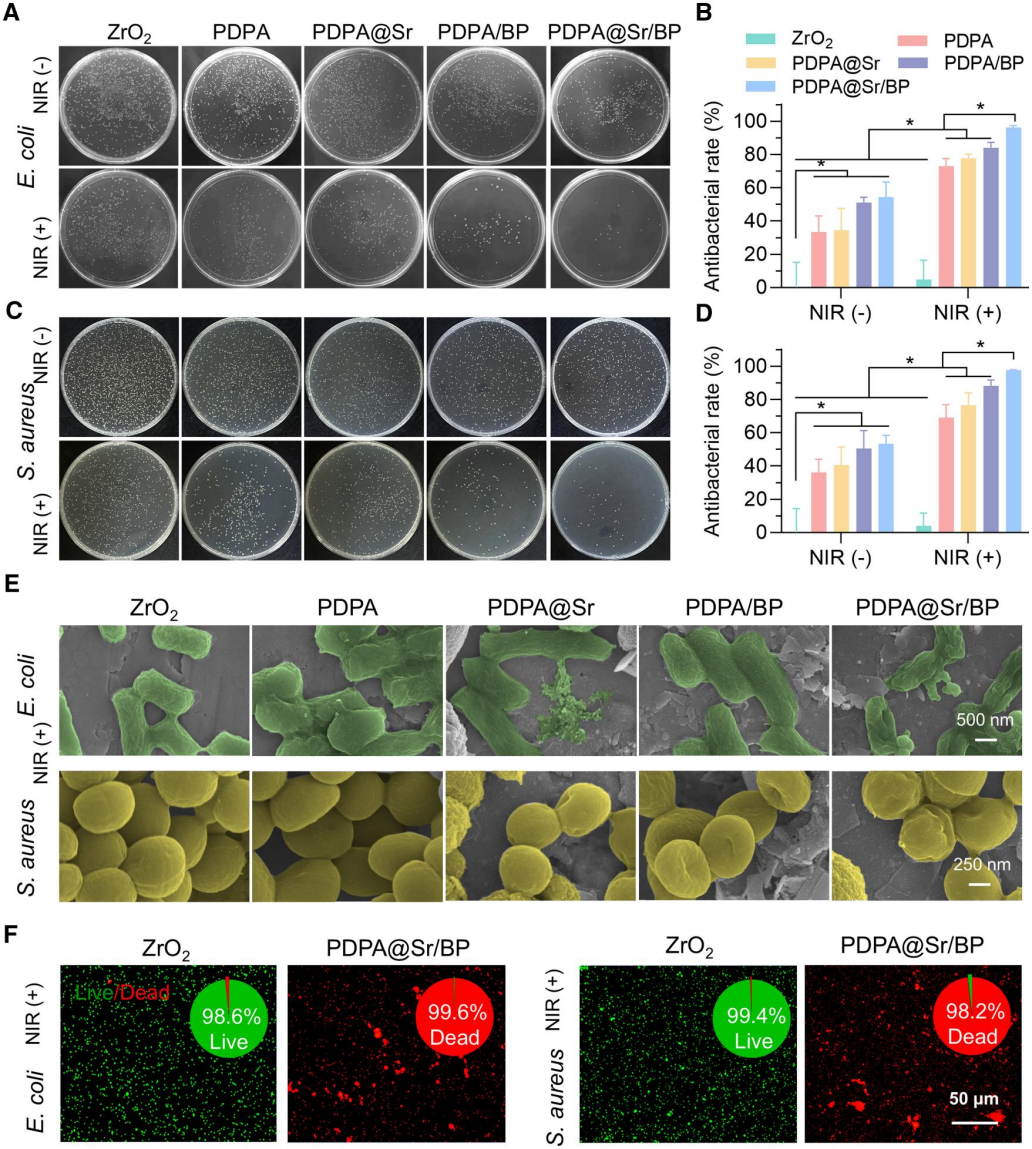
*In vitro* photothermal antibacterial properties. (**A**–**D**) Images of *S. aureus* and *E. coli* colonies from sample surfaces and the corresponding antibacterial rate. (**E**) SEM images of bacteria on the surface of each group of samples with NIR (+). (**F**) Live/dead assay images of bacterial colonies. Green and red indicate live and dead bacteria, respectively (*P < 0.05, *n* = 6).

In this experiment, two bacterial strains—*E. coli*, a gram-negative bacterium, and *S. aureus*, a gram-positive bacterium—were selected to evaluate the photothermal antibacterial performance of the PDPA@Sr/BP composite. These bacteria are commonly used in antibacterial experiments as major contributors to a wide range of bacterial infections [46–49]. The antibacterial test results against *E. coli* are shown in Figure 5A. Without NIR irradiation, all materials exhibited minimal inhibitory effects against both bacterial strains. The unmodified ZrO_2_ ceramic samples allowed extensive bacterial colony growth on LB agar plates, whereas modified coatings demonstrated a reduction in the number of viable colonies, with the PDPA@Sr/BP group exhibiting the lowest number of colonies. Upon 10 min of NIR irradiation, the number of viable colonies decreased significantly, and the PDPA@Sr/BP group maintained very few colonies on the LB agar plates. Antibacterial rates were calculated for each group (Figure 5B). Without NIR irradiation, PDPA@Sr/BP exhibited an antibacterial rate of 54.31%, slightly higher than that of other materials. Upon NIR irradiation, the PDPA, PDPA@Sr and PDPA/BP groups demonstrated moderate antibacterial efficacy, with rates of 72.84%, 77.59% and 84.05%, respectively. Notably, PDPA@Sr/BP achieved an antibacterial rate of 96.12%, indicating that the photothermal effect of the PDPA@Sr/BP coating effectively eradicated *E. coli* on the surface. Antibacterial testing against *S. aureus* yielded similar outcomes (Figure 5C). Regardless of NIR irradiation, the PDPA@Sr/BP group displayed significantly greater antibacterial efficiency compared to the other groups. The quantitative analysis (Figure 5D) revealed that PDPA, PDPA@Sr, PDPA/BP and PDPA@Sr/BP exhibited antibacterial rates exceeding 30% without NIR irradiation, while irradiation increased these rates to above 70%, indicating moderate antibacterial efficacy. The PDPA@Sr/BP group achieved an antibacterial rate of 97.57%. These results clearly show that NIR irradiation significantly enhanced the antibacterial effects of PDPA, PDPA@Sr, PDPA/BP and PDPA@Sr/BP materials, with PDPA@Sr/BP demonstrating the greatest improvement and optimal antibacterial performance against both *E. coli* and *S. aureus*. SEM images of bacterial surfaces after NIR irradiation (Figure 5E) provided further confirmation of these results. Bacteria on ZrO_2_ and PDPA surfaces appeared smooth with intact cellular structures, indicating weak antibacterial properties for unmodified and PDPA-modified ZrO_2_. In contrast, bacteria on PDPA@Sr and PDPA/BP surfaces exhibited slight surface roughness, whereas bacterial cells on the PDPA@Sr/BP surface showed severe damage, including surface indentations and ruptures. This indicates that the PDPA@Sr/BP composite, co-doped with Sr^2+^ and BPNs, displayed superior antibacterial activity by effectively eradicating both *S. aureus* and *E. coli* through a synergistic photothermal mechanism. Additionally, live/dead bacterial staining was conducted to compare the antibacterial performance of the PDPA@Sr/BP coating and unmodified ZrO_2_ (Figure 5F). Live bacteria are shown in green, while dead bacteria are shown in red. The ZrO_2_ surface exhibited high bacterial survival rates (*E. coli*: 98.6%, *S. aureus*: 99.4%), indicating poor bactericidal efficacy. Conversely, the PDPA@Sr/BP surface displayed a high bacterial death rate (*E. coli*: 99.6%, *S. aureus*: 98.2%), demonstrating excellent bactericidal efficiency under NIR irradiation.

Although *S. aureus* and *E. coli* are common bacterial strains, they are not the primary oral pathogens. Future studies will focus on evaluating the coating’s performance against oral pathogenic bacteria. In summary, the *in vitro* antibacterial experiments demonstrated that the PDPA coating, which possesses certain photothermal conversion properties, exhibits antibacterial activity. After the adsorption of BPNs, the coating effectively suppresses bacterial growth on the sample surface due to its superior photothermal conversion performance, as evidenced by the disruption of bacterial morphology and significant reductions in bacterial survival rates. Notably, the incorporation of Sr^2+^ into the coating leads to an enhancement in its antibacterial performance. Previous experiments have shown that the degradation rate of BPNs decreases upon Sr^2+^ doping, indicating that the PDPA@Sr/BP composite can sustain strong photothermal antibacterial activity for over two weeks, thus achieving long-term antibacterial effects.

### Osteogenic characteristics *in vitro*

**Figure 6.**
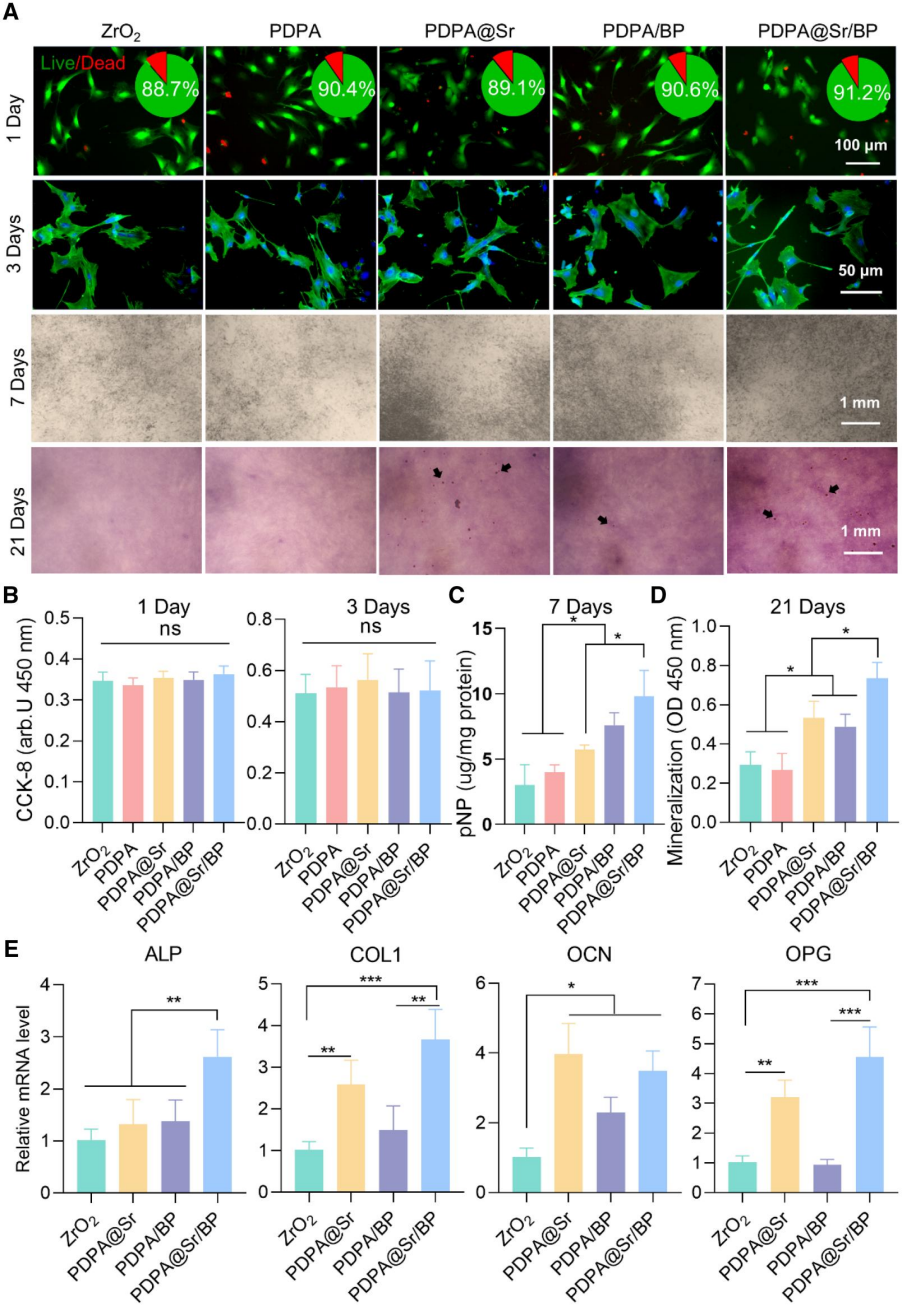
*In vitro* osteogenic performance. (**A**) Images of the live/dead assay (1 day), cell proliferation (3 days), ALP staining (7 days) and ARS staining (21 days) in different groups. Black arrows indicate the mineralized nodules. (**B**–**D**) Quantitative analysis of CCK-8 assay, ALP activity and Alizarin Red S staining in different groups. (**E**) Relative mRNA expression of ALP, COL1, OCN and OPG genes (*P < 0.05, **P < 0.01 and ***P < 0.001, *n* = 6).

Mouse pre-osteoblast MC3T3-E1 cells were cultured on the sample surfaces to assess the biocompatibility of the degradable coating. Live/dead staining images of cells after 1 day of culture (Figure 6A) indicated that the proportion of live cells exceeded 85% in all groups. After 3 days of culture, cells were stained with fluorescent dyes to visualize the cytoskeleton and nuclei to characterize MC3T3-E1 cell adhesion and morphology. Cells on the surfaces of all sample groups exhibited a typical and uniform morphology, with the extension of pseudopodia, indicating normal growth of MC3T3-E1 cells on the sample surfaces. The proliferation and viability of MC3T3-E1 cells after 1 and 3 days of culture were assessed by CCK-8 assay (Figure 6B). There were no significant differences in cell proliferation across the sample groups. In summary, the results indicate that the PDPA@Sr/BP coating does not disrupt the growth, adhesion or proliferation of MC3T3-E1 cells, demonstrating excellent biocompatibility.

To evaluate the effect of PDPA@Sr/BP on osteogenic differentiation of surface cells, we assessed early and late-stage osteogenesis in MC3T3-E1 cells and analysed the expression of osteogenesis-related genes. Osteogenic differentiation was initially evaluated by measuring ALP activity, an early marker indicative of osteoblast metabolic activity. As shown in Supplementary Figure S3A and Figure 6A, the PDPA@Sr, PDPA/BP and PDPA@Sr/BP groups exhibited significant ALP activity at 7 days, with the PDPA@Sr/BP group demonstrating the most pronounced effect. Quantitative analysis (Figure 6C) further supports this observation. A similar trend was observed in the late-stage osteogenic differentiation results. After 21 days of culture, ARS staining results (Figure 6A) revealed that the PDPA@Sr and PDPA@Sr/BP groups exhibited the highest number of mineralized nodules, while the PDPA/BP surface showed significantly fewer nodules in comparison. Quantitative analysis of the mineralization assay (Figure 6D) also indicated that cells on the PDPA@Sr/BP surface had the highest degree of mineralization, with PDPA/BP slightly lower than PDPA@Sr. This indicates that, in *in vitro* experiments, the BP coating alone does not have a significant effect on late-stage osteogenesis. Previous results (Figure 4A and B) indicated that BP in the PDPA@BP coating nearly completely degrades within one week, which may be one of the reasons for the coating’s excellent performance in early-stage osteogenesis and its decreased effectiveness in late-stage osteogenesis. However, PDPA@Sr/BP exhibited superior performance in both early and late-stage osteogenesis *in vitro*, likely attributable not only to the prolonged degradation rate of BP, but also to the synergistic osteogenic function by Sr^2+^ and ${\text{PO}}_{4}^{3-}$.

The expression levels of osteogenic-related genes in MC3T3-E1 cells cultured on the surface of the coatings were subsequently investigated (Figure 6E). ALP is an early marker of osteogenic differentiation, with upregulation of ALP gene expression indicating the initiation of osteogenic differentiation and suggesting the cells' capability for pre-mineralization [50]. COL1 is the primary component of the bone matrix, and increased expression of COL1 reflects substantial bone matrix synthesis, which marks the mid-stage of osteogenic differentiation and enhanced bone matrix formation [50, 51]. OCN, a non-collagenous protein secreted by osteoblasts, is typically expressed in the late stages of differentiation. Upregulation of OCN expression indicates the maturation of osteoblasts and promotion of mineralization and bone tissue formation [50, 52]. OPG is a regulatory protein involved in bone resorption and formation, acting to inhibit osteoclast generation and activity. Elevated OPG expression suggests that osteoblasts have acquired the ability to inhibit bone resorption while promoting bone formation during differentiation [53]. Collectively, the upregulation of these gene markers reflects the progression of MC3T3-E1 cells through various stages of osteogenic differentiation: early differentiation (ALP), mid-stage differentiation (COL1), late-stage differentiation (OCN) and mature osteoblast function regulating bone resorption (OPG). As expected, the PDPA@Sr/BP group demonstrated a significant enhancement in the mRNA expression of these osteogenic-related genes compared to the other groups. The PDPA@Sr group also exhibited relatively high mRNA expression levels, with a slightly higher OCN expression than the PDPA@Sr/BP group.

Consistent with the results from the ALP activity and mineralization assays, the upregulation of osteogenic-related genes in MC3T3-E1 cells cultured on the PDPA@Sr/BP surface indicates that the combination of Sr^2+^ and BP synergistically enhances the cells' osteogenic differentiation potential. Previous studies have shown that Sr^2+^ shares structural similarities with Ca^2+^, allowing Sr^2+^ to significantly enhance osteogenic properties through mechanisms involving the calcium-sensing receptor (CaSR) [43, 44]. Although BP is known to promote osteogenesis [37, 54–56], the PDPA/BP group did not exhibit a significant increase in osteogenic gene expression compared to the control group. This could be attributed to the instability of BP alone on the PDPA coating, as its rapid degradation may limit its expected osteogenic effects. However, when Sr^2+^ and BP are co-modified on the PDPA coating surface, the synergistic interaction enhances the stability of BP, resulting in a significant improvement in the osteogenic differentiation of MC3T3-E1 cells.

### *In vivo* evaluation of antibacterial

**Figure 7.**
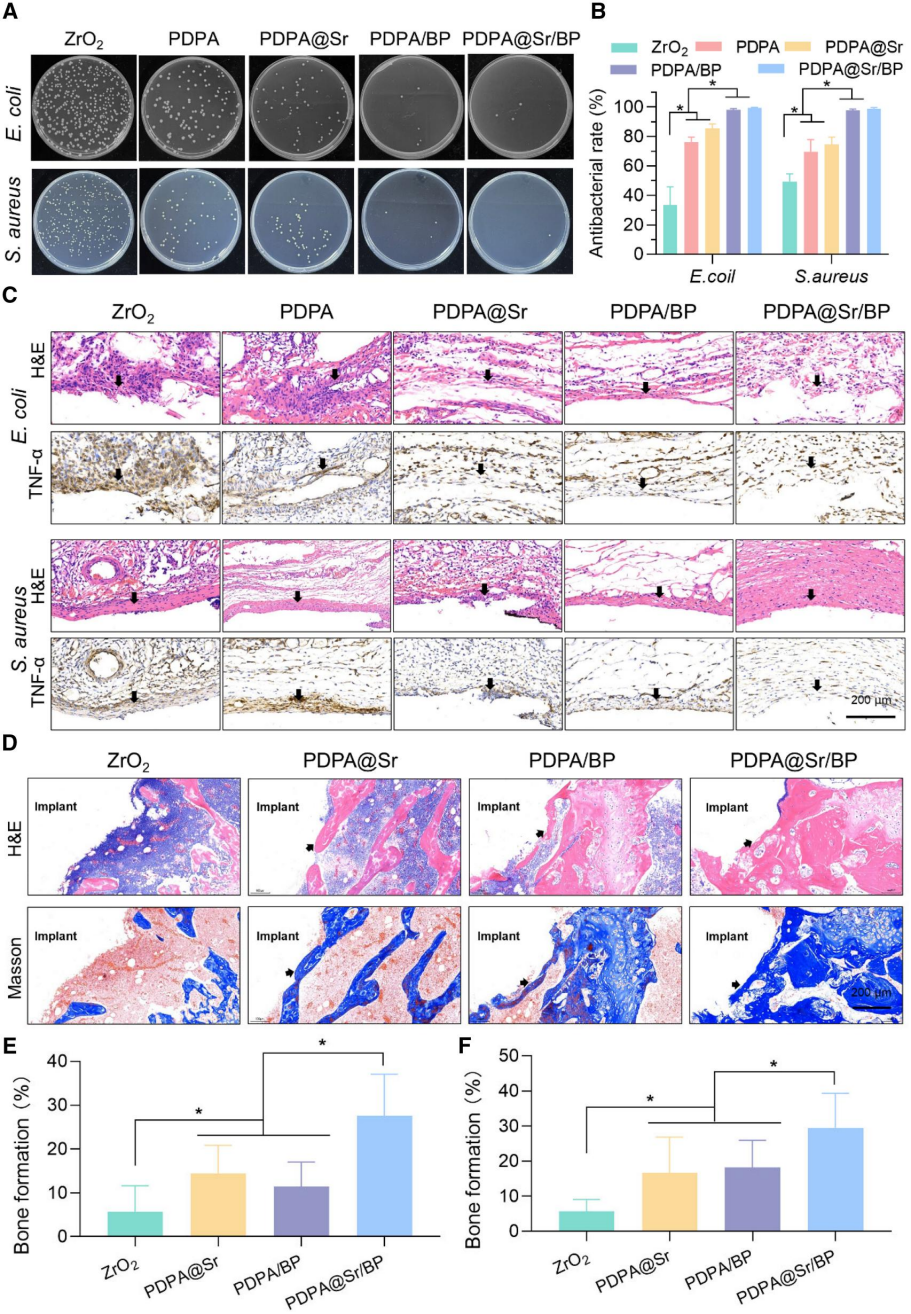
*In vivo* evaluation of antibacterial and osteogenic performance. (**A**) Representative culture images of *E. coli* and *S. aureus* colonies from sample surfaces under NIR (+) and (**B**) the corresponding antibacterial rate. (**C**) Images of H&E staining and TNF-α immunohistochemical staining in skin tissues. (**D**) Images of histological analysis of H&E and Masson staining and (**E**, **F**) the corresponding quantitative staining analysis (*P < 0.05, *n* = 4).

To evaluate the *in vivo* antibacterial efficacy of PDPA@Sr/BP, bacteria from the implant surfaces were transferred to LB agar for culture. As shown in Supplementary Figure S4A, under NIR (−) conditions, substantial bacterial colony growth was observed in the ZrO_2_ group, while viable colonies in the PDPA/BP and PDPA@Sr/BP groups were significantly reduced. After NIR irradiation (Figure 7A), the ZrO_2_ group continued to allow substantial bacterial colony growth, indicating the limited *in vivo* antibacterial capacity of unmodified ZrO_2_. In contrast, viable colonies on PDPA/BP and PDPA@Sr/BP were further reduced. This demonstrates that both coatings possess strong *in vivo* antibacterial properties, which were further enhanced under NIR irradiation. Based on the antibacterial efficiency calculations (Supplementary Figure S4B and Figure 7B), under non-irradiated conditions, PDPA/BP and PDPA@Sr/BP exhibited antibacterial efficiencies of 90.56% and 93.82% against *E. coli*, respectively. With NIR irradiation, these efficiencies increased to 97.99% and 99.42%. Similarly, colony counting for *S. aureus* showed antibacterial efficiencies of 92.93% and 94.46% without NIR irradiation. Following NIR irradiation, the antibacterial performance of both coatings was further enhanced, with PDPA/BP and PDPA@Sr/BP achieving nearly complete bacterial eradication, reaching efficiencies of 97.69% and 98.92%, respectively. Based on the *in vivo* antibacterial tests against both *E. coli* and *S. aureus*, it can be concluded that PDPA/BP and PDPA@Sr/BP exhibited distinct antibacterial effects against both bacterial strains *in vivo*. Moreover, NIR irradiation significantly enhanced the antibacterial efficacy of these coatings. Notably, PDPA@Sr/BP outperformed PDPA/BP, with antibacterial efficiency reaching approximately 99%, indicating a high level of antibacterial performance. These results suggest that the PDPA@Sr/BP composite coating has strong potential for preventing bacterial colonization on implant surfaces, thereby reducing the risk of infection and improving implant success rates.

To assess the extent of infection following implantation, tissue samples surrounding the ZrO_2_ implants from different groups were collected and analysed through histological examination. As shown in Figure 7C, severe bacterial infection resulted in significant infiltration of inflammatory cells in the soft tissues directly adjacent to the ZrO_2_ and PDPA ceramic pieces. The expression of TNF-α was notably elevated, indicating substantial release of inflammatory cytokines and a pronounced local inflammatory response. This suggests that the soft tissues surrounding the ZrO_2_ and PDPA groups experienced considerable inflammation. In contrast, the tissue structure around the PDPA@Sr group showed notable improvement, with a reduced inflammatory response. Particularly in the case of *S. aureus* infection, the tissue integrity was better preserved, and TNF-α expression was lower, indicating a milder inflammatory reaction. The soft tissues surrounding the PDPA/BP group also demonstrated improved tissue structure and moderate levels of inflammation. In both *E. coli* and *S. aureus* infections, the inflammatory response in the PDPA/BP group was better controlled, with cytokine expression significantly reduced compared to the ZrO_2_ and PDPA groups, indicating more effective management of inflammation. The PDPA@Sr/BP group exhibited the most intact tissue architecture in H&E staining, with the lowest level of inflammatory response. Notably, under *S. aureus* infection, there was minimal tissue irritation, and TNF-α expression was the lowest among all groups, indicating the mildest local inflammatory reaction. These findings demonstrate that the PDPA@Sr/BP composite exhibited the best anti-inflammatory performance of all materials tested.

### *In vivo* osteogenic performance

The macroscopic appearance, surface morphology and roughness of the ZrO_2_ implants were evaluated prior to implantation (Supplementary Figure S5), showing a smooth surface with no visible cracks or defects. To verify the *in vivo* osteogenic performance, implants were extracted from the rat femoral condyle one month after implantation, and the bone tissue surrounding the implants was subjected to histological examination. As shown in Figure 7D, the ZrO_2_ group exhibited a degree of inflammatory response around the implant in H&E staining, with a loose tissue structure, indicating poor integration between the bone tissue and the implant. Masson staining revealed minimal collagen fiber generation around the implant, suggesting limited osteogenic potential for ZrO_2_, which failed to significantly promote new bone formation. In contrast, the PDPA@Sr group demonstrated a milder cellular response, with a much tighter interface between the tissue and the implant, suggesting better early-stage osteogenesis. Masson staining revealed significant collagen fiber formation, with dense blue staining, indicating that PDPA@Sr effectively promoted new bone formation and exhibited favorable osteogenic performance. The PDPA/BP group displayed some cellular infiltration and slightly insufficient collagen fiber generation, demonstrating a moderate ability to promote new bone formation but falling short compared to PDPA@Sr. The PDPA@Sr/BP group showed the most favorable outcomes. H&E staining revealed the mildest inflammatory response, with minimal cellular infiltration and a clear interface between the bone tissue and the implant. Masson staining demonstrated the richest collagen fiber formation, with very prominent blue staining, indicating that PDPA@Sr/BP significantly promoted new bone formation and exhibited the best osteogenic performance among all groups. The quantitative analysis of H&E staining and Masson staining for each group further validated the above results (Figure 7E and F).

In physiological environments, the degradation products of BPNs can convert into calcium phosphate nanoparticles, which have the ability to promote bone regeneration through *in situ* biomineralization [18, 32]. Although these findings suggest that BP could play a beneficial role in bone defect treatments, BP-based therapeutic platforms rely on the presence of Ca^2+^ in the physiological environment to achieve biomineralization and bone regeneration [57]. Since an appropriate Ca/P ratio is crucial for bone regeneration, calcium-free BP-based strategies may be less effective in elderly patients with bone defects or those with bone damage due to Ca^2+^ loss [58, 59]. Sr^2+^ can partially replace Ca^2+^ without noticeable side effects, playing a crucial role in maintaining the Ca/P ratio. The combination of Sr^2+^ and BP in the PDPA@Sr/BP coating enables effective *in situ* biomineralization, which likely explains why PDPA@Sr/BP demonstrated superior osteogenic performance *in vivo* compared to the other groups.

It is noteworthy that the PDPA/BP group did not demonstrate as strong osteogenic promotion *in vivo* as it did *in vitro*. This discrepancy may be attributed to the rapid degradation of BP in the PDPA/BP coating, as evidenced in previous studies. The BP in the PDPA/BP group underwent significant degradation within 7 days, consistent with the cell cycle duration observed *in vitro*. However, *in vivo* experiments typically extend over a month, and the rapid degradation of BP may have limited its ability to support *in situ* mineralization throughout the entire experiment. On the other hand, BP degradation in the PDPA@Sr/BP group persisted beyond 14 days, allowing for the continuous release of physiologically relevant degradation products over most of the *in vivo* study, effectively promoting the mineralization of bone tissue surrounding the implant. Although 14 days is insufficient to cover the entire osteogenesis cycle, the excellent early osteogenic properties contribute positively to the final bone formation outcome [60].

Additionally, we deduced that the Sr^2+^ released directly from the implant coating not only promotes osteogenic differentiation of cells but also binds directly to ${\text{PO}}_{4}^{3-}$ at the implant-bone interface. This interaction may enhance the mineralization of bone in direct contact with the implant surface, facilitating the formation of thicker and denser new bone, as previously validated in animal studies.

In this study, the biodegradable PDPA@Sr/BP coating demonstrated superior osteogenic, antibacterial and stability properties compared to traditional Sr-doped coatings, BP composites and PDA-based coatings. First, this coating combines the synergistic effects of Sr^2+^ and BP degradation products, optimizing the Ca/P ratio and effectively promoting *in situ* biomineralization, addressing the limitations of Sr coatings that rely solely on ion release for osteogenic stimulation [61]. Second, compared to conventional antibiotic or silver nanoparticle-based antibacterial strategies, PDPA@Sr/BP utilizes BP’s photothermal effect for controllable antibacterial action, avoiding the risk of bacterial resistance while significantly enhancing antibacterial performance under NIR irradiation. Moreover, the incorporation of Sr^2+^ enhances BP stability and prolongs its degradation period to over 14 days, ensuring sustained bioactivity during bone integration. Unlike conventional PDPA-based coatings, which offer only a single bioactive modification, PDPA@Sr/BP provides strong adhesion, enhanced osteogenic potential and photothermal antibacterial functionality, making it a more promising candidate for dental implant applications. Therefore, this study extends the application of functionalized PDPA coatings in bone implants and proposes a dual-effect strategy combining osteogenic and antibacterial functions, offering a novel approach for optimizing bone integration materials.

## Conclusion

In summary, a degradable composite coating was successfully developed on the surface of ZrO_2_ implants by integrating Sr^2+^ and BPNs into the PDPA matrix. The physiological degradation of BPNs preserved the esthetic appearance of the ZrO_2_ implant, and its products (including ${\text{PO}}_{4}^{3-}$ and ${\text{HPO}}_{4}^{2-}$) exhibited significant synergistic osteogenic effects with Sr^2+^. Additionally, the incorporation of Sr^2+^ improved the stability of BP, extending its degradation period to over two weeks, effectively covering the critical early stage of osteogenesis post-implantation. *In vitro* results demonstrated that the degradable coating possesses excellent photothermal antibacterial properties, favorable biocompatibility, and promotes osteogenic differentiation in cells in both the short and long term. *In vivo* experiments, the coating’s prolonged degradation period, while maintaining excellent antibacterial properties, enabled a significant enhancement in osteogenic activity. Overall, the strategic combination of Sr^2+^ and BPNs not only enhanced the stability of the coating but also provided synergistic pathways for both antibacterial efficacy and osteogenesis at the implant–bone interface, making it an ideal multifunctional coating for ZrO_2_ implants.

## Supplementary Material

rbaf033_Supplementary_Data
