# Supplementary material for: Sr-doped surfaces with 2D black phosphorus nanosheets for enhanced photothermal antibacterial activity and zirconia implant osseointegration
Source: Regen Biomater. 2025 Apr 26;12:rbaf033. doi: 10.1093/rb/rbaf033 (PMC12116421; doi:10.1093/rb/rbaf033)
Supplement: rbaf033_Supplementary_Data [file rbaf033_supplementary_data.zip › Supplementary materials.docx]

**Supplementary materials**

**Sr-doped surfaces with 2D black phosphorus nanosheets for enhanced photothermal antibacterial activity and zirconia implant osseointegration**

Huan Cheng^1, †^, Jiaquan Chen^1, †^, Yan Wang^1^, Yinyan Zhang^1^, Tianyun Qin^1^, Haobo Sun^1^, Wen Si ^1^, Ningyao Sun ^1^, Yingyue Sun ^1^, Lifeng Xiong ^1^, Zhennan Deng^1, *^, Lei Lu^1, *^, Peng Gao^1, *^, Jinsong Liu^1^

1*. School and Hospital of Stomatology, Wenzhou Medical University, Wenzhou, 325027, China.*

†. *These authors contributed equally.*

****. Corresponding authors:***

*Zhennan Deng, dengzhennan@wmu.edu.cn;*

*Lei Lu,* [*llu2@foxmail.com*](mailto:llu2@foxmail.com)*;*

*Peng Gao, penggaocake@foxmail.com.*

**Table S1. Primers of target and housekeeping genes.**

| **Targets** | **Primers** |
| --- | --- |
| ALP | F:5′- GAACAGAACTGATGTGGAATACGAA -3′ |
|  | R:5′- CAGTGCGGTTCCAGACATAGTG -3′ |
| COL1 | F: 5′- GACATGTTCAGCTTTGTGGACCTC -3′ |
|  | R:5′- GGGACCCTTAGGCCATTGTGTA -3′ |
| OCN | F:5′- GAACAGACAAGTCCCACACAGC -3′ |
|  | R:5′- TCAGCAGAGTGAGCAGAAAGAT -3′ |
| OPG | F:5′- GCCCAGACGAGATTGAGAG -3′ |
|  | R:5′- CAGACTGTGGGTGACGGTT -3′ |
| GAPDH | F:5′- CTCGTCCCGTAGACAAAATGGT -3′ |
|  | R:5′- GAGGTCAATGAAGGGGTCGTT -3′ |


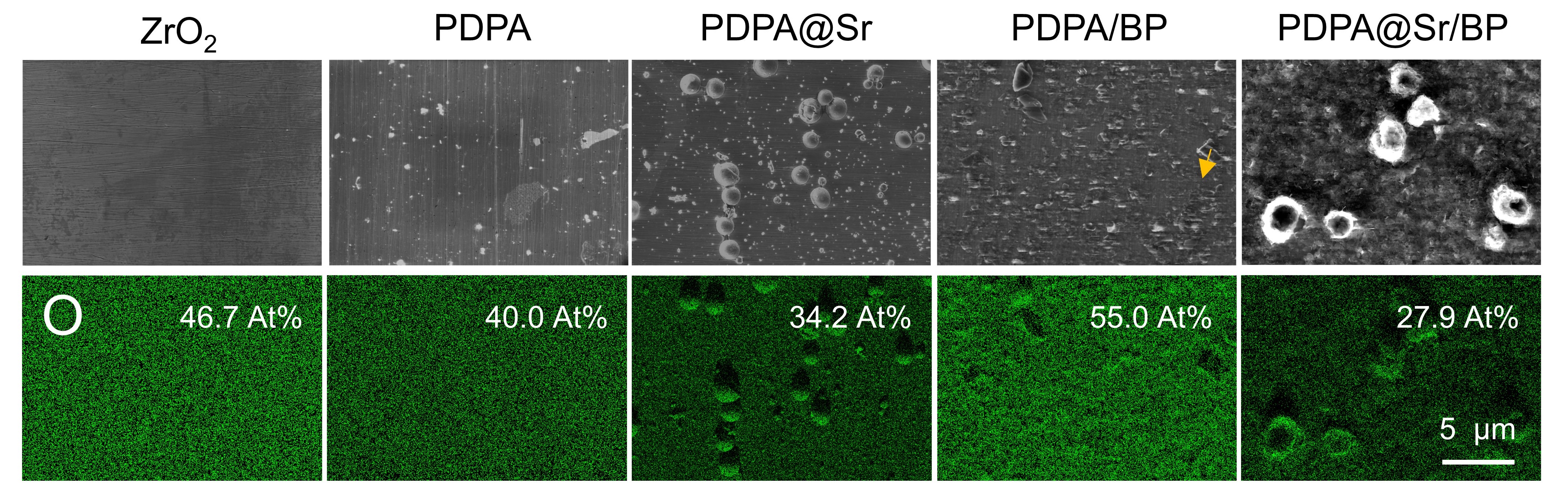


**Figure S1.** **Characterization of PDPA@Sr/BP Coating.** Representative SEM images of the sample surface and EDS mapping of the oxygen element.


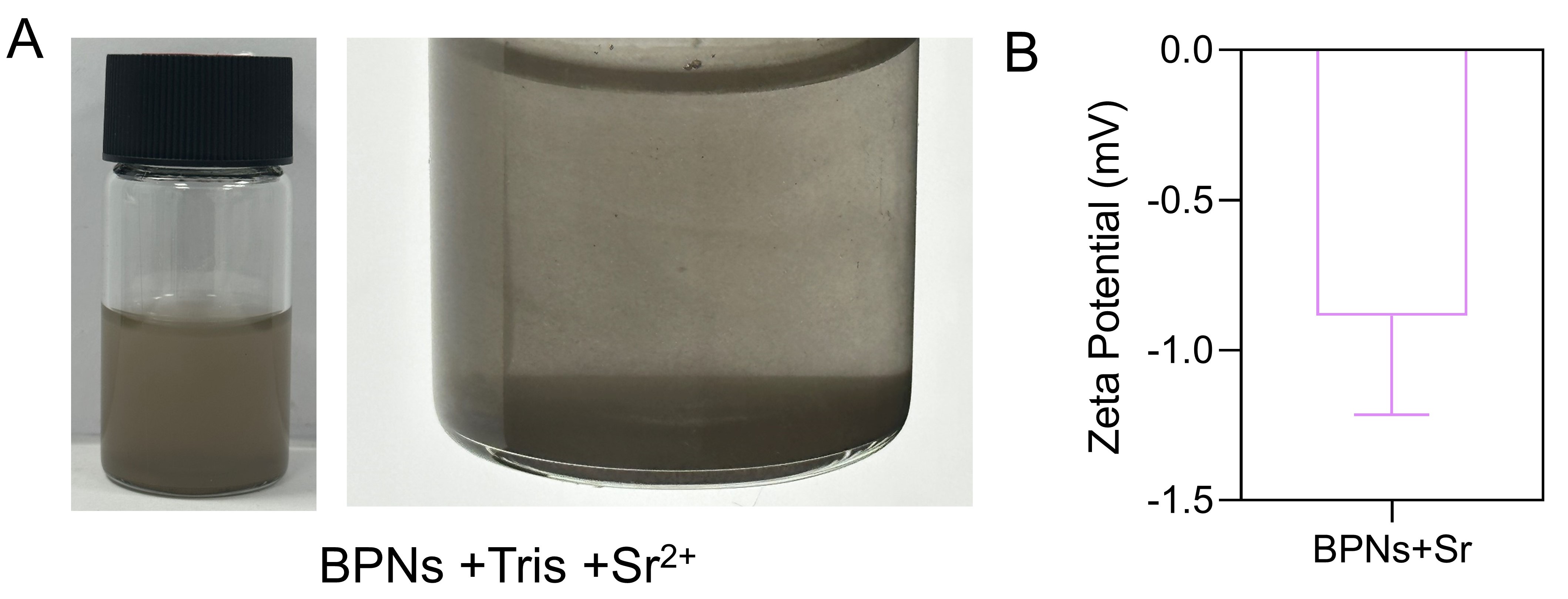


**Figure S2.** **Interaction between Sr²⁺ and BPNs.** (A) Images of the BPNS and Sr²⁺ mixed suspension. (B) The Zeta potential of the BPNS and Sr²⁺ mixed suspension. (n=3).


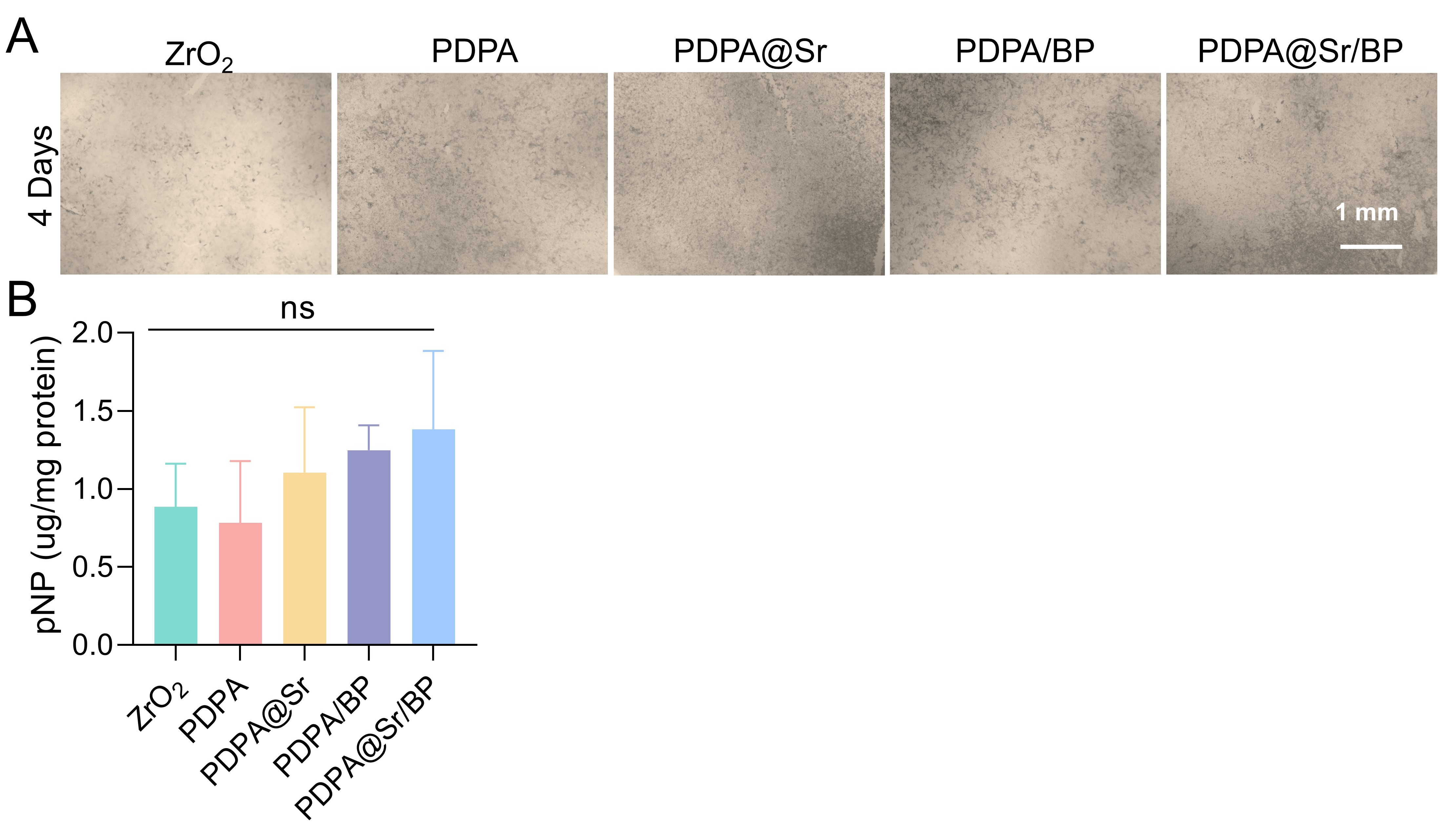


**Figure S3.** ***In vitro* osteogenic performance.** (A) Representative images of the ALP staining (4 d) in different groups (n = 6). (B) Quantitative analysis of ALP activity. “ns” (Non-significant), (n=4).


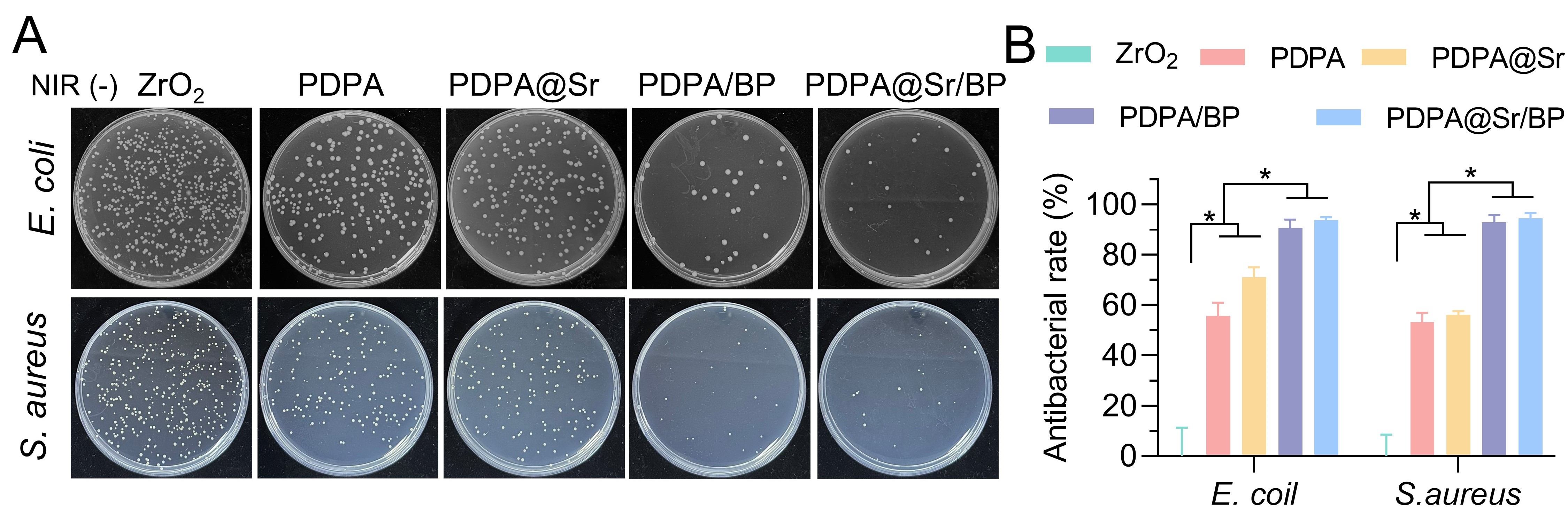


**Figure S4.** ***In vivo* evaluation of antibacterial and osteogenic performance.** (A) Representative culture images of *E. coli* and *S. aureus* colonies from sample surfaces under NIR (-). (B) Corresponding antibacterial rate. (*P <0.05), (n=4).


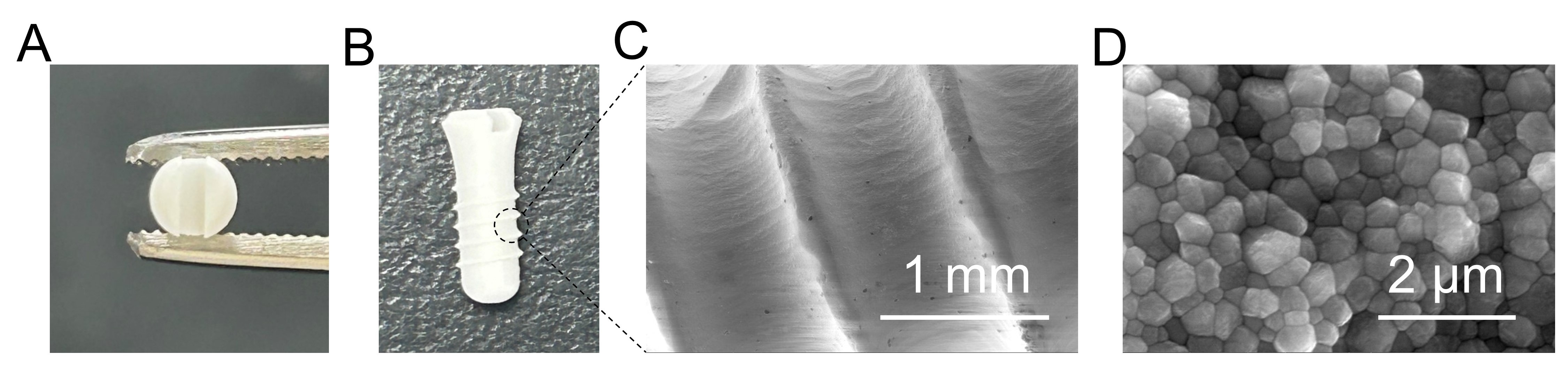


**Figure S5. Screws used in the *in vivo* experiment.** Photographs of the screw from the (A) top view and (B) front view. (C, D) SEM images of the screw surface.
